# Soluble triggering receptor expressed on myeloid cells-1 (sTREM-1) and other inflammatory mediators in malaria by *Plasmodium vivax* during enteroparasites coinfection

**DOI:** 10.1371/journal.pone.0270007

**Published:** 2022-06-24

**Authors:** Myrela Conceição Santos de Jesus, José Hugo Romão Barbosa, Rubens Alex de Oliveira Menezes, Margarete do Socorro Mendonça Gomes, Lays Gisele Santos Bomfim, Tamirys Simão Pimenta, Andrea Regina de Souza Baptista, Ricardo Luiz Dantas Machado, Tatiana Rodrigues de Moura, Luciane Moreno Storti-Melo

**Affiliations:** 1 Programa de Pós-Graduação em Microbiologia e Parasitologia Aplicadas, Instituto Biomédico, Universidade Federal Fluminense, Niterói, Rio de Janeiro, Brasil; 2 Programa de Pós-Graduação em Biologia Parasitária, Universidade Federal de Sergipe, São Cristóvão, Sergipe, Brasil; 3 Departamento de Ciências Biológicas e da Saúde, Universidade Federal do Amapá, Macapá, Amapá, Brasil; 4 Superintendência de Vigilância em Saúde do Estado do Amapá, Macapá, Amapá, Brasil; 5 Health Sciences Graduate Program, Federal University of Sergipe, São Cristóvão, Brazil; 6 Instituto Evandro Chagas / Secretaria de Vigilância em Saúde / Ministério da Saúde, Ananindeua, Pará, Brasil; 7 Departamento de Morfologia, Centro de Ciências Biológicas e da Saúde, Universidade Federal de Sergipe, São Cristóvão, Sergipe, Brasil; 8 Departamento de Biologia, Centro de Ciências Biológicas e da Saúde, Universidade Federal de Sergipe, São Cristóvão, Sergipe, Brasil; Para Federal University, BRAZIL

## Abstract

Malaria is a major health issue with more than 200 million cases occurring annually. Moreover, in Malaria endemic area are frequently observed Malaria-enteroparasite co-infections associated with the modulation of inflammatory response. In this aspect, biomarkers play an important role in the disease prognosis. This study aimed to evaluate inflammatory mediators in malaria during coinfection with enteroparasites. A subset of serum samples already collected was analyzed and divided into four groups: Malaria (n = 34), Co-infected (n = 116), Enteroparasite (n = 120) and Control (n = 95). The serum levels of sTREM-1 and IL-6 were measured by ELISA. TNF-α, and IL-10 levels were previously carried out by flow cytometry. Higher serum levels of sTREM-1 and IL-6 were showed in malaria patients compared to healthy controls. In co-infected malarial patients sTREM-1 serum levels were similar to control group. Interestingly, co-infected malaria patients showed IL-6 serum levels decreased compared to individuals only infected with *P*. *vivax*. However, in Malaria patients and co-infected there was a positive correlation between the IL-6 and IL-10 levels (P < 0.0001). This is the first report of sTREM-1 levels in *P*. *vivax* infected. Moreover, the results revealing a divergent effect of co-infection with the increased balance between pro-and anti-inflammatory cytokines and reduced IL-6 levels but increases the anemia occurrence. The results also highlight the potential use of IL-6 as a biomarker for *P*. *vivax* and enteroparasites coinfection.

## Introduction

Malaria is a major parasitic disease with high morbidity and mortality worldwide, with 228 million cases occurring only in the year 2018 [1]. In humans this disease is caused by five different species of *Plasmodium* [2] of which *P*. *vivax* is the species with greatest epidemiological interest as it is the most widely distributed species in regions outside of the African continent. *P*. *vivax* is responsible for high morbidity caused by the infection, and brings social and economic losses to the countries where this disease is present [3, 4]. Cases of severe malaria due to infection by this species have been described in the literature more frequently [5–8].

In 2018 more than 194,000 cases of the disease occurred in Brazil, of which more than 99% are from the Amazon region [9]. Another important health issue in the same area is the high circulation of enteroparasites [10–12] which, during a co-infection with *Plasmodium vivax*, may alter the immunopathological course. A previous study conducted by our group [13] analyzed the impact of co-infection *Plasmodium*-enteroparasite in the municipality of Oiapoque, located in a border area between Brazil and French Guiana. The results showed an increase in the levels of the cytokines TNF-α, and IL-10 in individuals co-infected with *P*. *vivax* and enteroparasitoses. However, this data raises new questions about the impact of other inflammatory immunological markers since it was not possible to verify the influence of co-infection on the cytokines profile that characterizes the Th1 and Th2 pattern.

Receptors present on the surface of innate immune cells can recognize components of microorganisms and initiate the inflammatory response. Among these, the Triggering Receptor Expressed on Myeloid Cells 1 (TREM-1) is a receptor expressed in macrophages, monocytes, and neutrophils that is known to increase inflammatory responses of myeloid cells in innate and adaptive responses that were initiated by the Toll-like receptors [14, 15]. The increase in serum levels of the soluble form of TREM-1 (sTREM-1) has been associated with more severe profiles in other parasitic diseases, such as visceral leishmaniasis [16], and has also been reported in malaria by *P*. *falciparum* [17–19].

In addition to sTREM-1, several cytokines have been described that play a role in controlling infection and symptom development [20]. Between them, TNF-α, interleukin 10 (IL-10) and IL-6 are molecules that can be a powerful inflammatory marker of severity during malaria. IL-6 has often been associated with increased severity during malaria caused by *P*. *vivax* in Brazil’s extra-amazon [21] and Amazon regions [22] however this cytokine has not yet been evaluated in this scenario of malaria coinfection with enteric pathogens.

This study therefore aims to evaluate the levels of inflammatory mediators in malarial patients and to compare with individuals co-infected with enteroparasites residents of the municipality of Oiapoque, Amapá state, North Brazil.

## Material and methods

### Subjects and sample collection

A subset of samples already collected was analyzed out of individuals previously evaluated by Menezes et al. [13]. The samples were collected between November 2014 and November 2015 in the municipality of Oiapoque, Amapá State, in northern Brazil. Only participants who were native to Oiapoque were included and signed a consent form to participate in the research study. Participants were over seven years of age and agreed to provide blood and stool samples.

Participants were divided into four groups: (1) Malaria: individuals with malaria by *P*. *vivax* who were negative for intestinal parasites (n = 34); (2) Co-infected: individuals with malaria and intestinal parasite co-infection (n = 116); (3) Enteroparasite: individuals without malaria and positive for an intestinal parasite (n = 120); (4) Control: individuals who were negative for malaria and intestinal parasite (n = 95). Blood collection was performed during diagnosis for malaria so none of the participants were using anti-malarial drugs to the time of blood collection. Ten millilitres of venous blood was collected from each patient by venipuncture. Four millilitres was dispensed into a tube with EDTA to perform haematological analysis and to prepare thick blood smear slides, and six millilitres was dispensed into a tube with no anticoagulant for immunological analysis. The serum samples were stored at -20°C in the Center for Microorganisms’ Investigation of the Fluminense Federal University until use. The diagnosis and pro and anti-inflammatory cytokine profiles was conducted according to the methodology described below [13].

Estimation of the hematological and parasitological parameters against *P*. *vivax* and enteroparasite infection.

The diagnosis for malaria, the quantification of parasitemia, the quantification of gametocytes, and hemoglobin dosage have been previously performed, as well as the enteroparasites diagnosis [13]. The diagnosis for malaria was made by microscopic analysis and confirmed by molecular diagnosis, following a previously described protocol [23]. The fecal diagnosis was performed using the technique and/or methods of Hoffman-Pons-Janer and Faust. For each sample, two slides were examined for detection of parasites by two investigators with identification experience, using optical microscopy (Nikon, Japan) with magnifications of 100X and 400X. For the negative cases, three fecal samples were requested on alternate days to increase the detection sensitivity.

In addition, hemoglobin dosage was performed according to the protocol recommended by the World Health Organization through hematocrit index. The hematological parameters evaluated were the total number of erythrocytes (RBC; reference range: male 4.5–6.5 x 106/μL, female 3.9–5.6 x 106/μL and children aged 7–11 years 4.5–4.7 x 106/μL) and hemoglobin levels (Hb; males 13 g/dL, females 12 g/dLand children 11 g/dL). Individuals were considered anemic when their haemoglobin blood levels were 13 g/dL for males, 12 g/dL for females, and 11 g/dL for the children.

### Assessment of TNF-α, and IL-10 response against *P*. *vivax*

Cytokine levels were detected by flow cytometry according to previously published guidelines in the plasma samples [13]. Quantification of cytokines was conducted using a cytometric bead array kit (BD). A standard curve was performed for each cytokine and analyzed using FACSDiva software (Becton Dickinson, San Jose, CA, USA). The bead populations were displayed according to their respective fluorescence intensities, from dimmer to brighter. In the CBA, the cytokine capture beads were mixed with detection antibody conjugated with PE fluorochrome and then incubated with the test samples to form a "sandwich" test. The acquisition tubes were prepared with 50 μL of the sample, 50 μL of bead mix, and 50 μL of Th1/Th2 PE detection reagent (Human Th1/Th2 PE Detection Reagent/1 vial, 4 mL). The results were presented on graphs and in tables using FCAP Array 3 software (Becton Dickinson, San Jose, CA, USA). Raw MFI (median fluorescence intensity) values were quantified for each cytokine. The values were expressed in pg/mL for each cytokine in comparison to the standard curve. Three hundred events were considered for each cytokine.

### Quantification of sTREM-1 and IL-6 by ELISA

In addition to the existing cytokine panel, IL-6 and sTREM-1 levels were also dosed. The levels of sTREM-1 in the serum were measured by using Human TREM-1 DuoSet ELISA kit (R&D Systems, Minneapolis, MN, EUA) and IL-6 levels were measured by IL-6 Human Uncoated ELISA Kit (Thermo Fisher Scientific, Waltham, MA, EUA). The absorbance was measured using a microplate reader Epoch (BioTek, Winooski, VT, EUA) at 450 nm and a wavelength correction at 570 nm to subtract the background. A standard curve was generated for each plate using the manufacturer’s recommended protocol.

### Statistical analysis

Differences between the two groups were calculated using the Mann–Whitney U-test, or the Kruskal-Wallis test with the Dunn showing mean and standard error. Differences with p < 0.05 were considered statistically significant. Analyses were performed using Prism 8.4.3 software (GraphPad).

### Ethical considerations

The samples included in this study were collected with the approval of the Research Ethics Committee of the Federal University of Amapá. The participants who agreed to participate signed a written informed consent form. For minor participants, their parents or guardians signed the consent forms.

This study was also approved by the Ethics Committee of the Fluminense Federal University (CAAE number 40790120.7.0000.0003) to reuse the samples and waiving the consent of the participants and parents or guardians of the minors for this new analysis.

## Results

**Fig 1:**
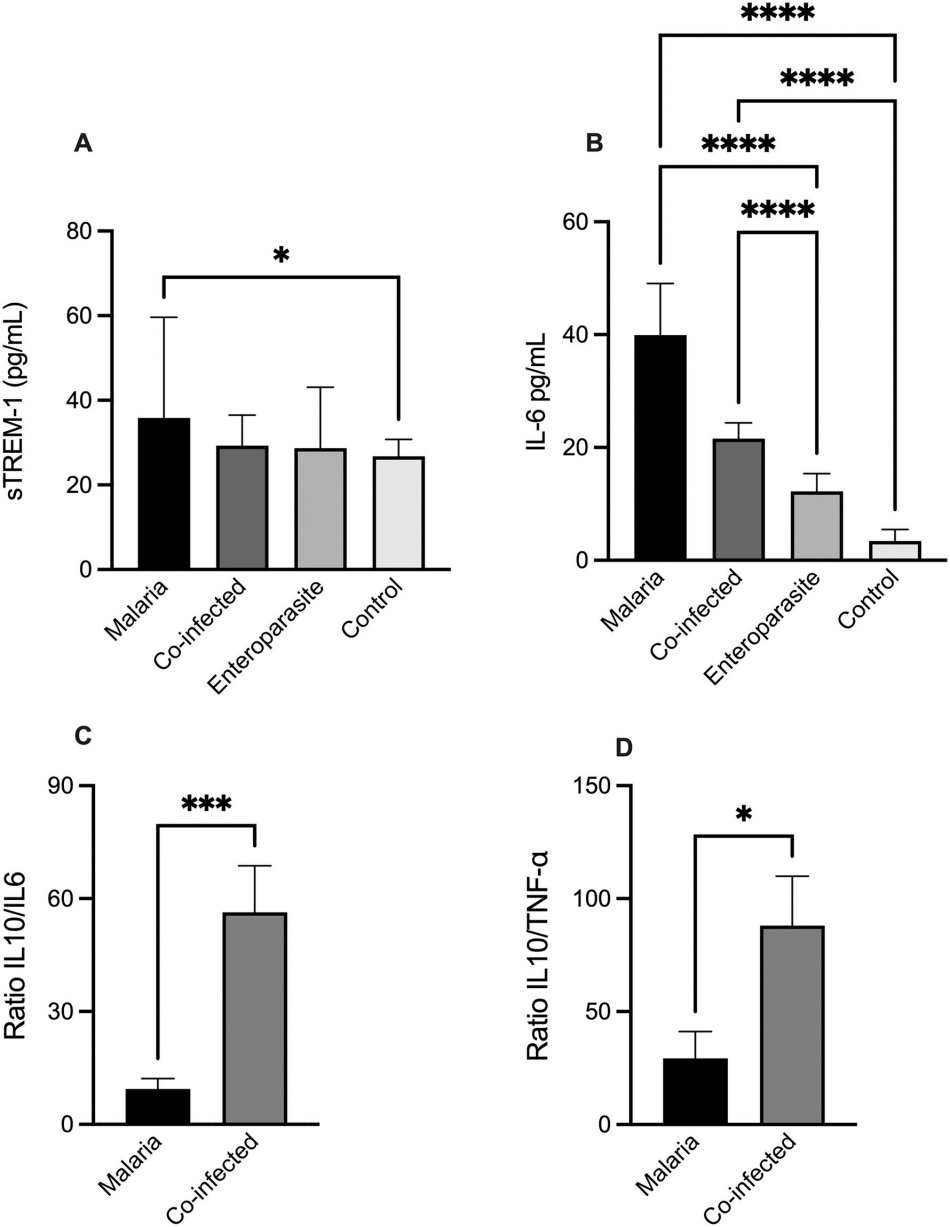
Differential levels of inflammatory mediators during malaria with sTREM-1 and IL-6 serum levels were associated with malaria infection and IL-6 serum levels were also associated during enteroparasites co-infection. **(A)** sTREM-1 measured in serum samples from malaria-infected individuals (n = 34), co-infected patients (n = 116), enteroparasite patients (n = 120) and healthy controls (n = 95). Bars represent the mean ± standard error (*P < 0.05, by Mann Whitney test). The asterisk represents a statistically significant difference between the malaria and the control and group. **(B)** IL-6 measure in serum samples from malaria-infected individuals (black; n = 34), co-infected patients (blue; n = 116), enteroparasite patients (purple, n = 120) and healthy controls (pink, n = 95). Bars represent the mean ± standard error (****P < 0.0001, by Mann Whitney test). The asterisk represents a statistically significant difference between the malaria and the control and group. **(C)** The ratio between the levels of IL-10 and IL-6 in serum samples from malaria-infected individuals (n = 34) and co-infected patients (n = 116). Bars represent the mean ± standard error (***P < 0.001, by Mann Whitney test). The asterisk represents a statistically significant difference between the malaria and the control and co-infected group. **(D)** Ratio between the levels of IL-10 and TNF-α in serum samples from malaria infected (n = 34) and co-infected individuals (n = 116). Bars represent the mean ± standard error (*P < 0.05, by Mann Whitney test). The asterisk represents a statistically significant difference between the malaria and the control and co-infected group.

Serum levels of sTREM-1 and IL-6 were measured in patients with malaria and controls. Considering the high prevalence of enteroparasitosis circulating in the region and that this infection can alter the immune course of malaria this was also measured the levels in two other comparison groups: co-infected and enteroparasites. Results showed a higher level of sTREM-1 and IL-6 in individuals with malaria compared with individuals without the infection (P < 0.05). The level of sTREM-1 was similar between the groups, although a decreasing trend was seen for the co-infected group (Fig 1A). Interestingly for IL-6, in the malaria group the level was 6.9 higher than control (P < 0.0001) and the results showed higher levels of this cytokine compared to all other groups (P < 0.005). In the co-infected group, the IL-6 levels were higher than in the control group (P < 0.002) (Fig 1B).

Given the importance of the pro-and anti-inflammatory balance during malaria, a comparison was performed between the ratio of the main pro-inflammatory (IL-6 and TNF-α) and anti-inflammatory (IL-10) cytokines identified in the malaria and coinfected groups. In this analysis, the coinfected group had a higher level of IL-10 than the inflammatory cytokines, compared to the profile seen in the malaria group (Fig 1C and 1D).

**Fig 2:**
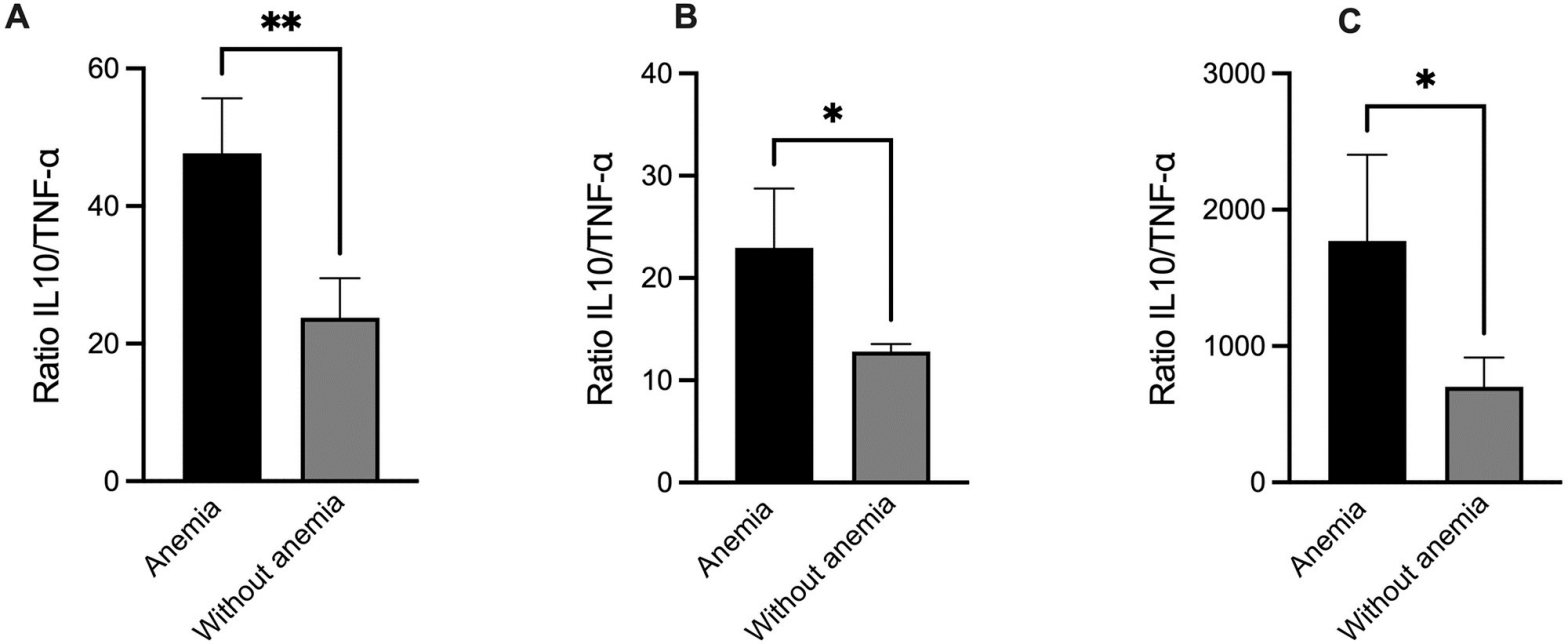
Higher levels of inflammatory mediators were correlated with anemia in patients coinfected with *P*. *vivax* and enteroparasites. **(A)** IL-6 levels in serum samples from co-infected patients with anemia (n = 15) and without anemia (n = 48). Bars represent the mean ± standard error (*P < 0.05, by Mann Whitney test). The asterisk represents a statistically significant difference between the malaria and the control and co-infected group. **(B)** TNF-α levels in serum samples from co-infected patients with anemia (n = 15) and without anemia (n = 48). Bars represent the mean ± standard error (*P < 0.05, by Mann Whitney test). The asterisk represents a statistically significant difference between the malaria and the control and co-infected group. **(C)** IL-10 levels in serum samples from co-infected patients with anemia (n = 15) and without anemia (n = 48). Bars represent the mean ± standard error (*P < 0.05, by Mann Whitney test). The asterisk represents a statistically significant difference between the malaria and the control and co-infected group.

Considering previous findings that these samples showed an increase in anemia in the coinfected group [13], an evaluation of the three main inflammatory mediators identified in the coinfected group with and without anemia was carried out to identify if there is a difference in the levels of these cytokines in these groups. It was observed from this analysis that the group of individuals with anemia had higher levels of IL-6, TNF- α, and IL-10 cytokines than the individuals without anemia (P < 0.05) (Fig 2A–2C).

## Discussion

The inflammatory response generated during malaria is essential for parasite clearance however enhanced inflammation can trigger immunopathological damage to the host [24, 25]. This is the first study that describes the levels of sTREM-1 in individuals infected with *P*. *vivax* and has identified significantly increased levels in infected individuals (P < 0.05).

The sTREM-1, the soluble portion of TREM-1, has been extensively investigated as a potential biomarker of severity [16, 20, 26–28]. In parasitic infections, high levels of sTREM-1 were observed in patients with Visceral Leishmaniasis who died, and the levels of sTREM-1 were negatively correlated with leukocyte count and hemoglobin concentration and were associated with increased parasitemia [16]. Similarly, a study conducted in Uganda with patients infected with malaria by *P*. *falciparum* identified sTREM-1 as a new biomarker of severe and fatal malaria in children with this malaria profile [26].

Our study is thus the first to include only non-severe patients infected by *P*. *vivax* which is the most widely distributed species of *Plasmodium* worldwide [1, 3] and observe a difference in sTREM-1 levels when compared to healthy controls. However, when comparing with the coinfected and enteroparasite groups no significant difference was seen, although a decreasing trend in the coinfected group was observed. This may be due to the fact that all the individuals included here had mild symptoms and the main findings already described with sTREM-1 and parasitic diseases correlate this protein with a severity prognosis [16, 20, 26].

Furthermore, experimental studies of sTREM-1 induction with LPS in animal models demonstrated that when low-dose LPS (2 ng/kg) was used, the increase in sTREM-1 was transient and decreased progressively within the first 24 hours [29]. If the same concept applies for malaria, the low parasitemia should influence the data found here considering that many samples have not detected sTREM-1 levels. It must be considered that the serum used in this analysis was collected and refrigerated between 2014 and 2015 years and the sTREM-1 in some samples may have degraded over time. Therefore, the results described here could differ if they are made with fresh samples, we acknowledge.

Our results also demonstrated that IL-6 serum levels increased 6.9 times in malaria patients compared to healthy individuals. In addition, IL-6 was associated positively with IL-10, which is compatible with the mild forms in the patients included in this study. Athough, higher IL-6 production during *P*. *falciparum* infection was observed to be associated with increased clinical episodes [30], the IL-6 levels do not change the memory response development in *P*. *vivax*, since no variations were observed in the production of anti-PvMSP-1_19_ IgG antibodies in our previous study with these same samples [13].

In contrast to what was found for sTREM, IL-6 levels were significanty lower in the coinfected group compared to the malaria group. The malaria group showed higher values of this cytokine compared with all other groups, demonstrating that IL-6 levels are highly stimulated during *P*. *vivax* infection. Interestingly, the difference observed between malaria and the coinfected group seems to indicate a modulation of inflammation in individuals coinfected with enteroparasites. As expected, the IL-10/IL-6 ratio was lower in malaria group when compared to the coinfected, showing that in the coinfected the response was leaning more towards a suppressive profile. This may indicate a potential protective effect of co-infection on malaria by *P*. *vivax*. In malaria by *P*. *falciparum*, this protective effect has already been seen in acute renal failure and jaundice [31] and co-infection with *Ascaris lumbricoides* was protective against cerebral malaria [32]. A study conducted in Africa with people coinfected with *P*. *falciparum* and soil borne helminth, coinfection led to increased IL-10 and IL-6 levels and a down-regulation of TGF-β [33]. In our study with *P*. *vivax*, the coinfected had increased IL-10/IL-6 and IL-10/TNF-alpha ratios and decreased IL-6 levels when compared to patients who were infected with malaria infection only.

However, another possible scenario must be considered. In the acute phase of infection, a predominantly pro-inflammatory response is important for parasitemia control and parasite clearance [34]. Given that all the individuals were included in the study at the time of diagnosis it is likely that most of them were in the acute phase and therefore this immunoregulation may be favorable to the parasite, ensuring maintenance of the infection.

Additionally, increased anemia was seen in the coinfected compared to the malaria group [13] which may support the hypothesis that the suppressive profile may not be beneficial for the patients. Anemia during malaria is a multifactorial etiology. The incidence and severity increase in specific groups such as children under 5 years old, pregnant women, and can also be caused due to resistance during anti-malarial treatment, hemolysis, malnutrition, and other associated infections such as HIV, parvovirus B19, Intestinal helminths [35]. However, among these characteristics, only enteroparasite coinfection was present in the individuals included in our study.

It is worth of noting that despite the decrease in IL-6, this coinfected group showed higher values of TNF-α than malaria [13]. TNF-α is an important pro-inflammatory cytokine and during malaria has been correlated with the development of anemia [36]. The anemia in these patients may thus be developed as a result of the inflammatory response of these cytokines and the generation of a damage-associated molecular pattern (DAMPs) [37, 38] and/or due to a strong TH2 response that favors the persistence of the parasite and is generated by the natural cycle of *Plasmodium* replication that leads to red blood cells lysis.

As expected, in malaria patients, parasitemia was positively correlated with the anti and pro-inflammatory cytokines IL-10 and TNF-α. In a study conducted in a municipality in Amazonas, Brazil increased IL-10 and IL-6 were associated with parasite load [22]. In our study, although there was a tendency to increase, no significant association was found. For sTREM-1 no association with the cytokine panel or with the clinical variables parasitemia, gametocytes, and hemoglobin levels has been identified.

We also demonstrated a potential modulation of IL-6 during coinfection with intestinal parasites. It is important to mention that most malaria-endemic regions have a high prevalence of enteroparasites [31, 32, 39]. Nevertheless, few studies have focused on assessing the impact of co-infection on malaria prognosis. One limitation that we acknowledge is the inability to separate enteroparasites into the helminth, protozoan, and both groups in our analysis due to the low number of individuals in the groups.

Therefore, studies are needed to determine the impact of these immune molecules on each of these groups, especially sTREM-1 which has been scarcely studied in these populations. Moreover, studies have demonstrated the impact of polymorphisms in the *TREM-1* gene on the susceptibility, inflammatory role and prognosis of malaria and other diseases [17, 40–44]. It is also necessary therefore to assess the genetic variability of *TREM-1* in populations of endemic areas and their impact on the course of malaria by *P*. *vivax*. Finally, is the coinfection with enteroparasites beneficial during *P*. *vivax* infection in non-severe cases, and is the anemia developed during coinfection caused by immune damage or due to parasite persistence? are two key questions that need further clarification.

## Supporting information

S1 Data (XLSX)

## References

[1] WHO. World Malaria Report 2019. 2019 [Cited 2021 June 17]. https://www.who.int/malaria/publications/world-malaria-report-2019/en/.

[2] Phillips MA, Burrows JN, Manyando C, van Huijsduijnen RH, Van Voorhis WC, Wells TNC. Malaria. Nat Rev Dis Primers. 2017;3: 17050. doi: 10.1038/nrdp.2017.50 28770814

[3] WHO. World Malaria report 2018. 2018 [Cited 2021 June 17]. https://www.who.int/malaria/publications/world-malaria-report-2018/en/.

[4] Sarma N, Patouillard E, Cibulskis RE, Arcand J. The Economic Burden of Malaria: Revisiting the Evidence. Am J Trop Med Hyg. 2019;101: 1405–1415. doi: 10.4269/ajtmh.19-0386 31628735 PMC6896867

[5] Izri A, Cojean S, Leblanc C, Cohen Y, Bouchaud O, Durand R. *Plasmodium vivax* severe imported malaria in two migrants in France. Malar J. 2019;18: 422. doi: 10.1186/s12936-019-3067-5 31842880 PMC6916050

[6] Baird JK. "Lively" invasive *Plasmodium vivax* causes severe and complicated malaria. Travel Med Infect Di. 2019;30: 7–8. doi: 10.1016/j.tmaid.2019.06.004 31202701

[7] Im JH, Kwon HY, Baek J, Park SW, Durey A, Lee KH, et al. Severe *Plasmodium vivax* infection in Korea. Malar J. 2017;16: 51. doi: 10.1186/s12936-017-1684-4 28129766 PMC5273855

[8] Tatura SNN, Wowor EC, Mandei JM, Wilar R, Warouw SM, Rompis J, et al. Case Report: Severe *Plasmodium vivax* Malaria Mimicking Sepsis in a Neonate. Am J Trop Med Hyg. 2018;98: 656–659. doi: 10.4269/ajtmh.17-0739 29313481 PMC5930913

[9] Ministério da Saúde. Malária—Descrição da Doença. 2018 [Cited 2021 June 17]. http://portalms.saude.gov.br/saude-de-a-z/malaria/descricao-da-doenca.

[10] Gonçalves AQ, Junqueira ACV, Abellana R, Barrio PCD, Terrazas WCM, Sodré FC, et al. Prevalence of intestinal parasites and risk factors forspecific and multiple helminth infections in a remote city of the Brazilian Amazon. Rev. Soc. Bras. Med. Trop. 2016;49: 1–6. doi: 10.1590/0037-8682-0128-2015 27163576

[11] Confalonieri UEC, Margonari C, Quintão AF. Environmental change and the dynamics of parasitic diseases in the Amazon. Acta Trop. 2014;129: 33–41. doi: 10.1016/j.actatropica.2013.09.013 24056199

[12] Maia MMM, Fausto MA, Vieira ELM, Benetton MLFN, Carneiro M. Intestinal parasitic infection and associated risk factors, among children presenting at outpatient clinics in Manaus, Amazonas state, Brazil. Ann Trop Med Parasitol. 2009;103: 583–591. doi: 10.1179/000349809X12459740922417 19825280

[13] Menezes RAO, Gomes MSM, Mendes AM, Couto AARD, Nacher M, Pimenta TS, et al. Enteroparasite and vivax malaria co-infection on the Brazil-French Guiana border: Epidemiological, haematological and immunological aspects. PLoS One. 2018; 13: e0189958. doi: 10.1371/journal.pone.0189958 29293589 PMC5749708

[14] Bouchon A, Dietrich J, Colonna M. Cutting edge: inflammatory responses can be triggered by TREM-1, a novel receptor expressed on neutrophils and monocytes. J. Immunol. 2000;164: 4991–4995. doi: 10.4049/jimmunol.164.10.4991 10799849

[15] Bouchon A, Facchetti F, Weigand MA, Colonna M. TREM-1 amplifies inflammation and is a crucial mediator of septic shock. Nature. 2001;410: 1103–1107. doi: 10.1038/35074114 11323674

[16] Bomfim LGS, Magalhães LS, Santos-Filho MAA, Peres NTA, Corrêa CB, Tanajura DM, et al. *Leishmania infantum* Induces the Release of sTREM-1 in Visceral Leishmaniasis. Front Microbiol. 2017;8: 2265. doi: 10.3389/fmicb.2017.02265 29201022 PMC5696592

[17] Adukpo S, Gyan BA, Ofori MF, Dodoo D, Velavan TP, Meyer CG. Triggering receptor expressed on myeloid cells 1 (TREM-1) and cytokine gene variants in complicated and uncomplicated malaria. Trop Med Int Health. 2016;21: 1592–1601. doi: 10.1111/tmi.12787 27671831

[18] Bruneel F, Tubach F, Mira J, Houze S, Gibot S, Huisse M, et al. Imported falciparum malaria in adults: host- and parasite-related factors associated with severity. The French prospective multicenter PALUREA cohort study. Intensive Care Med. 2016;42: 1588–1596. doi: 10.1007/s00134-016-4356-x 27169586

[19] Conroy AL, Hawkes M, McDonald CR, Kim H, Higgins SJ, Barker KR, et al. Host Biomarkers are Associated with Response to Therapy and Long-Term Mortality in Pediatric Severe Malaria. Open Forum Infect Dis. 2016 Jun 20;3: 134. doi: 10.1093/ofid/ofw134 27703996 PMC5047396

[20] Antonelli LR, Junqueira C, Vinetz JM, Golenbock DT, Ferreira MU, Gazzinelli RT. The immunology of *Plasmodium vivax* malaria. Immunol Rev. 2020 Jan;293: 163–189. doi: 10.1111/imr.12816 31642531

[21] Ribeiro BP, Cassiano GC, Souza RM, Cysne DN, Grisotto MAG, Santos APSA, et al. Polymorphisms in *Plasmodium vivax* Circumsporozoite Protein (CSP) Influence Parasite Burden and Cytokine Balance in a Pre-Amazon Endemic Area from Brazil. PLoS Negl Trop Dis. 2016 Mar 4;10: e0004479. doi: 10.1371/journal.pntd.0004479 26943639 PMC4778932

[22] Costa AG, Antonelli LRV, Costa PAC, Pimentel JPD, Garcia NP, Tarragô AM, et al. The robust and modulated biomarker network elicited by the *Plasmodium vivax* infection is mainly mediated by the IL-6/IL-10 axis and is associated with the parasite load. J Immunol Res. 2014;2014: 318250. doi: 10.1155/2014/318250 24741587 PMC3987793

[23] Snounou G, Viriyakosol S, Zhu XP, Jarra W, Pinheiro L, Rosario VE, et al. High sensitivity of detection of human malaria parasites by the use of nested polymerase chain reaction. Mol Biochem Parasitol.1993;61: 315–20. doi: 10.1016/0166-6851(93)90077-b 8264734

[24] Gowda DC, Wu X. Parasite Recognition and Signaling Mechanisms in Innate Immune Responses to Malaria. Front Immunol. 2018;9: 1–17. doi: 10.3389/fimmu.2018.03006 30619355 PMC6305727

[25] Gazzinelli RT, Kalantari P, Fitzgerald KA, Golenbock DT. Innate sensing of malaria parasites. Nat. Rev. 2014;14: 744–757. doi: 10.1038/nri3742 25324127

[26] Erdman LK, Dhabangi A, Musoke C, Conroy AL, Hawkes M, Higgins S, et al. Combinations of host biomarkers predict mortality among Ugandan children with severe malaria: a retrospective case-control study. PLoS One. 2011;6: e17440. doi: 10.1371/journal.pone.0017440 21364762 PMC3045453

[27] Balanza N, Erice C, Ngai M, Varo R, Kain K, Bassat Q. Host-Based Prognostic Biomarkers to Improve Risk Stratification and Outcome of Febrile Children in Low- and Middle-Income Countries. Front Pediatr. 2020 Sep 18;8: 552083. doi: 10.3389/fped.2020.552083 33072673 PMC7530621

[28] Feng J, Su W, Pan S, Yeh Y, Lin Y, Chen N. Role of TREM-1 in pulmonary tuberculosis patients- analysis of serum soluble TREM-1 levels. Sci Rep. 2018 May 29;8: 8223. doi: 10.1038/s41598-018-26478-2 29844416 PMC5974358

[29] Jolly L, Carrasco K, Salcedo-Magguilli M, et al. sTREM-1 is a specific biomarker of TREM-1 pathway activation. Cell Mol Immunol. 2021;18(8): 2054–2056. doi: 10.1038/s41423-021-00733-5 34282296 PMC8322270

[30] Robinson LJ, D’Ombrain MC, Stanisic DI, Taraika J, Bernard N, Richards JS, et al. Cellular tumor necrosis factor, gamma interferon, and interleukin-6 responses as correlates of immunity and risk of clinical *Plasmodium falciparum* malaria in children from Papua New Guinea. Infect Immun. 2009 Jul;77: 3033–43. doi: 10.1128/IAI.00211-09 19380468 PMC2708537

[31] Nacher M, Singhasivanon P, Silachamroon U, Treeprasertsuk S, Vannaphan S, Traore B, et al. Helminth infections are associated with protection from malaria-related acute renal failure and jaundice in Thailand. Am J Trop Med Hyg. 2001 Dec;65: 834–836. doi: 10.4269/ajtmh.2001.65.834 11791982

[32] Nacher M, Gay F, Singhasivanon P, Krudsood S, Treeprasertsuk S, Mazier D, et al. *Ascaris lumbricoides* infection is associated with protection from cerebral malaria. Parasite Immunol. 2000 Mar;22: 107–113. doi: 10.1046/j.1365-3024.2000.00284.x 10672191

[33] Bwanika R, Kato CD, Welishe J, et al. Cytokine profiles among patients co-infected with *Plasmodium falciparum* malaria and soil borne helminths attending Kampala International University Teaching Hospital, in Uganda. Allergy Asthma Clin Immunol. 2018;14(10): 1–9. doi: 10.1186/s13223-018-0235-z 29560020 PMC5858126

[34] Wammes LJ, Wiria AE, Toenhake CG, Hamid F, Liu KY, Suryani H, et al. Asymptomatic plasmodial infection is associated with increased tumor necrosis factor receptor II-expressing regulatory T cells and suppressed type 2 immune responses. J Infect Dis. 2013 May 15;207: 1590–1599. doi: 10.1093/infdis/jit058 23408847

[35] Tavares JC. Malária. In: Colloquium Series on Integrated Systems Physiology: From Molecule to Function to Disease. 2013.

[36] Tchinda VH, Tadem AD, Tako EA, Tene G, Fogako J, Nyonglema P, et al. Severe malaria in Cameroonian children: correlation between plasma levels of three soluble inducible adhesion molecules and TNF-alpha. Acta Trop. 2007 Apr;102: 20–28. doi: 10.1016/j.actatropica.2007.02.011 17397790

[37] Wajant H, Siegmund D. TNFR1 and TNFR2 in the Control of the Life and Death Balance of Macrophages. Front Cell Dev Biol. 2019 May 29;7: 91. doi: 10.3389/fcell.2019.00091 31192209 PMC6548990

[38] Collins MK, Shotland AM, Wade MF, Atif SM, Richards DK, Torres-Llompart M, et al. A role for TNF-α in alveolar macrophage damage-associated molecular pattern release. JCI Insight. 2020 May 7;5: e134356. doi: 10.1172/jci.insight.134356 32255768 PMC7253030

[39] Menezes RAO, Gomes MSM, Mendes AM, Nascimento SR, Couto ÁARD, Nacher M, et al. High Frequency of Enteroparasitoses in the Municipality of Oiapoque, Amapá State, Brazil, on the Border with French Guiana. J Biomed Res Rev. 2019;2: 05–11. doi: 10.1101/627109

[40] Golovkin AS, Ponasenko AV, Yuzhalin AE, Salakhov RR, Khutornaya MV, Kutikhin AG, et al. An association between single nucleotide polymorphisms within TLR and TREM-1 genes and infective endocarditis. Cytokine. 2015;71: 16–21. doi: 10.1016/j.cyto.2014.08.001 25213166

[41] Rivera-Chávez FA, Huebinger RM, Burris A, Liu M, Minei JP, Hunt JL, et al. A TREM-1 Polymorphism A/T within the Exon 2 Is Associated with Pneumonia in Burn-Injured Patients. ISRN Inflamm. 2013 Feb 12;2013: 431739. doi: 10.1155/2013/431739 24049659 PMC3767327

[42] Tammaro A, Kers J, Emal D, Stroo I, Teske GJD, Butter LM, et al. Effect of TREM-1 blockade and single nucleotide variants in experimental renal injury and kidney transplantation. Sci Rep. 2016 Dec 8;6: 38275. doi: 10.1038/srep38275 27928159 PMC5143803

[43] Golovkin AS, Ponasenko AV, Khutornaya MV, Kutikhin AG, Salakhov RR, Yuzhalin AE, et al. Association of TLR and TREM-1 gene polymorphisms with risk of coronary artery disease in a Russian population. Gene. 2014 Oct 15;550: 101–109. doi: 10.1016/j.gene.2014.08.022 25128583

[44] Santo JE, Mesquita TGR, Silva LDO, Araújo FJ, Souza JL, Lacerda TC, et al. *TREM1 rs2234237* (Thr25Ser) Polymorphism in Patients with Cutaneous Leishmaniasis Caused by Leishmania guyanensis: A Case-Control Study in the State of Amazonas, Brazil. Pathogens. 2021 Apr 20;10: 498. doi: 10.3390/pathogens10040498 33924130 PMC8074324

